# Evidence of endometrial amino acid metabolism and transport modulation by peri-ovulatory endocrine profiles driving uterine receptivity

**DOI:** 10.1186/s40104-017-0185-1

**Published:** 2017-06-15

**Authors:** Moana Rodrigues França, Maressa Izabel Santos da Silva, Guilherme Pugliesi, Veerle Van Hoeck, Mario Binelli

**Affiliations:** 1 Department of Animal Reproduction, School of Veterinary Medicine and Animal Science, University of São Paulo, 225, Duque de Caxias Norte Ave. Jd. Elite, 13635-900 Pirassununga, SP Brazil; 2 Gamete Research Centre, University of Antwerp, Antwerp, Belgium

**Keywords:** Amino acids, Beef cattle, Sex steroids, Uterus

## Abstract

**Background:**

In beef cattle, changes in the periovulatory endocrine milieu are associated with fertility and conceptus growth. A large preovulatory follicle (POF) and the resulting elevated concentrations of progesterone (P4) during diestrus positively affect pregnancy rates. Amino acids (AA) are important components of maternally derived secretions that are crucial for embryonic survival before implantation. The hypothesis is that the size of the POF and the concentration of P4 in early diestrus modulate the endometrial abundance of SLC transcripts related to AA transport and metabolism and subsequently impact luminal concentrations of AA. The follicle growth of Nelore cows was manipulated to produce two experimental groups: large POF and CL (LF-LCL group) and small POF and CL (SF-SCL group). On Day 4 (D4; Experiment 1) and Day 7 (D7; Experiment 2) after GnRH-induced ovulation (GnRH treatment = D0), the animals were slaughtered and uterine tissues and uterine washings were collected. qRT-PCR was used to evaluate the expression levels of AA transporters in D4 and D7 endometrial tissues. The concentrations of AA were quantified in D4 and D7 uterine washings by HPLC.

**Results:**

Transcript results show that, on D4, *SLC6A6, SLC7A4, SLC17A5, SLC38A1, SLC38A7* and *SCLY* and on D7 *SLC1A4, SLC6A1, SLC6A14, SLC7A4, SLC7A7, SLC7A8, SLC17A5, SLC38A1, SLC38A7, SLC43A2* and *DDO* were more abundant in the endometria of cows from the LF-LCL group (*P* < 0.05). In addition, concentrations of AA in the uterine lumen were influenced by the endocrine profiles of the mother. In this context, D4 uterine washings revealed that greater concentrations of taurine, alanine and α-aminobutyric acid were present in SF-SCL (*P* < 0.05). In contrast, lower concentrations of valine and cystathionine were quantified on D7 uterine washings from SF-SCL cows (*P* < 0.05).

**Conclusion:**

The present study revealed an association between the abundance of transcripts related to AA transport and metabolism in the endometrium and specific periovulatory endocrine profiles related to the receptive status of the mother. Such insights suggest that AAs are involved in uterine function to support embryo development.

## Background

A profitable beef cattle production system requires high reproductive efficiency. Acceptable female fertility rates depend on an in-depth understanding of endocrine, cellular and molecular mechanisms regulating pregnancy. Hormonal variations during each bovine estrous cycle induce uterine changes that are crucial for uterine receptivity for conceptus development and implantation. Elevated levels of plasmatic progesterone (P4) immediately after conception are related to advanced conceptus elongation [1–3]. Moreover, recent studies have shown that low plasmatic concentrations of P4 are associated with a suboptimal uterine environment for conceptus (or blastocyst) development [3–5]. Conversely, a positive association exists between the probability of a successful pregnancy and plasma concentrations of P4 at D7 after estrus in both dairy and beef cows [4, 6]. However, the identities of molecules and mechanisms responsible for triggering the latter phenomena need further investigation.

In this context, information on the involvement of amino acid (AA) transport and metabolic pathways and availability to the embryo during early diestrus remains limited. During the first two weeks of pregnancy, before implantation, the conceptus depends exclusively on the intrauterine milieu created by the endometrial secretions or molecules transported into the lumen by the uterine endometrium prior to implantation and placentation [7]. Amino acids are important components of these maternally derived secretions that are crucial for embryonic survival mainly during early pregnancy [8–12]. Optimal amounts of essential and non-essential AAs are important for embryonic development, which is altered in case of suboptimal amounts of these molecules [13–15]. The regulation of AA transport and concentration in cells and tissues, including the placenta and endometrium, depends on specific transport proteins [16]. There is no consistent information regarding the regulation of protein synthesis and the activity of AA transporters; however, changes in transcriptional profiles of AA transporters reveal changes in AA availability in the uterine lumen [12]. Interestingly, AA transporter gene expression increases simultaneously in maternal endometrium and conceptus cells. This indicates transport of AAs from the maternal circulation to the endometrial lumen exclusively for embryonic development.

Amino acids seem to be indispensable for embryonic survival and development. When non-essential AAs were completely removed from the medium, blastocyst growth drastically decreased [14]; the same was observed in medium with any combination of glucose and phosphate without AAs [15]. More interestingly, P4 infusion in animals with no CL and no follicle increased AA availability in the uterus and maternal plasma of cows [17] and gene expression of AA transporters in ovine and bovine endometria [8, 12, 18].

During early embryonic development in vitro, AA requirements seem to change [19]. Until the blastocyst stage, an excess of essential AAs seems to be detrimental for embryonic development, at least in vitro [13]; however, the absence of non-essential AAs in the culture medium is unfavorable for embryonic development [14]. During the blastocyst expansion stage, few changes were observed in AA turnover during embryo culture [20], when patterns of AA depletion and release were similar to those observed in vitro for pre-elongation embryos [21]. More interestingly, AA turnover has been identified as an indicator of embryonic viability in humans and cattle [22, 23]. Another relevant finding is that enzymes present in endometrial tissue can regulate AA synthesis or degradation. These enzymes are not characterized in the bovine endometrium. In other tissues, DDO (D-aspartate oxidase) catalyzes the deamination of alanine and aspartate [24]. SCLY (selenocysteine lyase) is involved in the production of alanine and elemental selenium from selenocysteine [25].

In this context, AA uterine transport and luminal availability emerge as important variables that might play a role in maternal and embryonic well-being. However, there is limited information available on how changes in the pre- and post-ovulatory endocrine milieu affect the transport of AAs from the maternal circulation to endometrial cells and finally to the uterine lumen. We hypothesize that the size of the POF and subsequent physiological circulating concentrations of P4 in early diestrus modulate endometrial expression of AA transporter protein pathways.

We recently described a model to manipulate preovulatory follicle growth to produce groups of cyclic beef cows with distinctly different circulating preovulatory concentrations of E2 and early diestrus concentrations of P4 [26]. Based on the contrasting ovarian and endocrine characteristics of these two groups of animals, we studied the following variables on both D4 and D7 of the estrous cycle: (1) transcript abundance of AA transporters in endometrial tissues; (2) transcript abundance of enzymes related to AA metabolism in endometrial tissues; and (3) uterine luminal concentrations of AA. Previous studies indicated that the phenotype of smaller follicles and short proestrus was associated with lower receptivity and capacity to support conceptus development in comparison with a group manipulated to have a longer proestrus and ovulate a larger follicle [27]. We focused on D4 and D7 for our investigation because it is around D4-5 that the embryo moves from the oviduct to the uterus; thus, from this moment until implantation (i.e., D20 in cattle) the embryo depends exclusively on endometrial secretions for its development [28–30]. Moreover, most embryonic losses occur during the two first weeks of pregnancy [31]. Therefore, mechanistic insights retrieved from this animal model could serve as a potential basis for future strategies to fine-tune maternal receptivity towards the embryo.

## Methods

### Animals and reproductive management

**Fig. 1:**
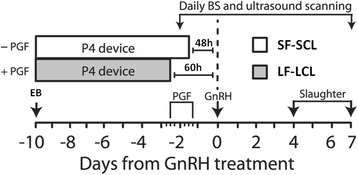
Experimental model and hormonal treatments. Growth of the pre-ovulatory follicle (POF) of beef cows was programmed to generate two groups of cows, the large follicle-large CL group (LF-LCL; associated with greater receptivity to the embryo and greater fertility) and small follicle-small CL group (SF-SCL). To decrease exposure to P4 and thereby stimulate growth of the POF, animals from LF-LCL group received an injection of PGF at the moment of intravaginal P4-releasing device insertion vs. no injections in the animals from SF-SCL group. Also, removal of the P4-releasing device was 12 h earlier in the LF-LCL group. Follicle size, ovulation and CL size were accessed by ultrasound scanning of the ovaries. Blood samples were collected for P4 assay. Ovulation was induced by GnRH on D0. On D4 (Experiment 1) and D7 (Experiment 2) animal were slaughtered for samples collection. BS, blood sampling; GnRH, 1 μg of busereline acetate im; P4, P4 progesterone-releasing device containing 1 g of P4; +PGF, cows received 0.5 mg of sodium cloprostenol on D-10; -PGF, cows did not receive Cloprostenol on D-10; EB, 2 mg of estradiol benzoate; Slaughter, endpoint for endometrial tissue and uterine washings collection (Adapted from Reference 26)

Eighty-six Nelore (*Bos indicus*) cows started in the experiment. The females selected were cycling, pluriparous, and non-lactating and did not present any detectable reproductive disorder. To form two distinct groups of females with different POF sizes and subsequent CL volumes and plasmatic concentrations of P4 (Fig. 1), a hormonal protocol was used as described previously [26, 27, 32].

Briefly, the cows received two injections of prostaglandin F2α (PGF; 0.5 mg; Cloprostenol; Sincrocio®; Ourofino, Cravinhos, SP, Brazil) 14 d apart. Next, the ovaries were examined using transrectal ultrasonography (US) to confirm the presence of a PGF-induced CL 10 d after the second PGF administration, (D − 10; D0 = day of induction of ovulation by GnRH injection). On D − 10, each female was treated with 2 mg of estradiol benzoate (Sincrodiol®, Ourofino, Cravinhos, SP, Brazil) to stimulate the emergence of a follicular wave and received an intravaginal P4-releasing device (1 mg; Sincrogest®; Ourofino, Cravinhos, SP, Brazil). Additionally, females assigned to the large POF followed by large CL (LF-LCL) group also received a PGF injection (0.5 mg; Cloprostenol; Sincrocio®) on D-10 to induce CL regression during follicle development, whereas cows assigned to the small POF followed by small CL (SF-SCL) group did not. Sixty hours prior to the induction of ovulation, the P4 devices were removed and an injection of PGF was administered to the LF-LCL females, whereas cows in the SF-SCL received PGF administration 12 h later (D − 2.5 and D − 2, respectively). Ovulation was induced on D0 by administration of a gonadotrophin-releasing hormone agonist (GnRH; 1 μg buserelin; Sincroforte®; Ourofino, Cravinhos, SP, Brazil).

Follicular growth, ovulation and CL diameter were monitored by transrectal ultrasound. Only cows that ovulated in response to the GnRH (i.e., between 24 and 36 h after GnRH injection; *N* = 57) were included in the data analysis. The cows were slaughtered and their reproductive tracts collected for further analysis on D4 (Experiment 1; LF-LCL n = 16; SF-SCL n = 8) or D7 (Experiment 2; LF-LCL n = 18 SF-SCL n = 18).

### Ultrasound Exams and CL Measurements

Transrectal ultrasound exams were performed using an Aloka SSD-500 device attached to a 5 MHz linear probe. The presence of an active CL on D-10, the time of ovulation, the size of the CL and the properties of the dominant follicle and POF were noted. Postmortem CLs were dissected and weighed separately, and the length, width and height were recorded. A calculated diameter for each CL was estimated by the mean of these three values. The CL volume was estimated using the formula for the volume of a sphere (V = 4/3πR^3^), where R was equal to half the mean diameter.

### Plasma Progesterone Measurement

Blood was collected from the jugular vein on D4 and D7. The blood samples were centrifuged at 1500 × g for 30 min at 4 °C. Plasma aliquots were stored at −20 °C until analysis. Plasmatic concentrations of P4 were measured by radioimmunoassay analysis (Coat-A-Count®; Siemens Medical Solutions Diagnostics, Munich, Germany) as described previously [33].

### Endometrial tissue and uterine fluid collection

Postmortem uteri were collected, and the uterine horns ipsilateral to the CL were washed with 20 mL PBS. Subsequently, the washings were centrifuged (1000 × g) for 30 min at 4 °C, and the supernatant was collected, snap frozen in liquid nitrogen and stored at -80 °C. Subsequently, endometrial tissue from the washed uteri was dissected from the intercaruncular region of the ipsilateral uterine horn, frozen in liquid nitrogen and stored at -80 °C.

### Quantification of amino acids

The concentrations of free AA in uterine flushings were determined by high-performance liquid chromatography (HPLC) using an HPLC Luna Column 3u C18(2) 100A 250 × 4.6 mm (00G-4251-E0 - Phenomenex, Torrance, CA, USA). The protocol for AA quantification was adapted from [34]. Briefly, 100 μL of internal standards of free AAs and methanol were added to 200 μg of uterine flushing samples (n = 12 LF-LCL, n = 7 SF-SCL for Experiment 1; n = 9 LF-LCL, n = 10 SF-SCL for Experiment 2). The dissolved samples were deproteinized using Vivaspin 500 (MWCO 3000 Da, Sartorius, Goettingen, Germany) followed by centrifugation (15,000×g for 45 min at 4 °C) and derivatization and were subsequently analyzed by programmed chromatography. The samples were analyzed in simplicate. The results were obtained by comparing each AA peak to its corresponding peak on a multilevel (3 levels) standard curve, based on certified standards.

### Analysis of gene expression levels in endometrial tissue

Total RNA was extracted from endometrial samples using the RNeasy Mini Kit (Qiagen Laboratories, Germantown, MD, USA) following manufacturer instructions. The concentration and purity of mRNA were estimated using NanoVue (GE Healthcare Life Sciences, Buckinghamshire, England). Total RNA extracts were stored at −80 °C until cDNA synthesis. One microgram of total RNA was used for reverse transcription using the High-Capacity cDNA Reverse Transcription kit (Life Technologies, Carlsbad, CA, USA), according to manufacturer’s instructions.

Quantification of endometrial gene expression was obtained by qPCR analysis using a StepOne Plus® apparatus from Applied Biosystems. Transcript abundance was determined for several AA-transport-related genes. The genes tested were *SLC1A1, SLC1A4*, *SLC1A5*, *SLC6A1, SLC6A6, SLC6A14*, SLC7A2*, SLC7A4*, *SLC7A5*, *SLC7A7, SLC7A8, SLC7A11,* SLC17A5, SLC17A9, *SLC36A2*, *SLC38A1*, *SLC38A4, SLC38A6, SLC38A7, SLC43A2, SCLY,* and *DDO*. Each primer pair was analyzed, considering the probabilities of hairpin, homodimer and heterodimer formation, using Oligo Analyzer 3.1 software (IDT®; http://www.idtdna.com/analyzer/Applications/OligoAnalyzer/). Sequence specificities were tested using the software *Basic Local Alignment Search Tool* (Blast) (http://blast.ncbi.nlm.nih.gov). PCR product identity was confirmed by sequencing (Table 1). Transcript abundance was compared between tissues from animals in the LF-LCL and SF-SCL groups from Experiment 1 (*n* = 8 per group) and Experiment 2 (*n* = 8 for LF-LCL and *n* = 9 for SF-SCL group). The selection of reference genes was performed using geNorm software (www.qbaseplus.com) [35]. After selection performed by the software, RPS18 was selected as an endogenous control for endometrium on D4, and cyclophilin, β-actin and GAPDH were chosen for endometrium on D7. Transcript abundance was calculated as the relative abundance between the target gene and the geometric average of selected housekeeping genes and given as an arbitrary value.

**Table 1:** Target genes, primer sequence and amplicon information

Gene	Gene Symbol	Representative ID	Sequence	Amplicon size, bp
Solute Carrier Family 1 member 1	*SLC1A1* ^a^	NM_174599.2	F: AAGGAGTTGGAGCAAATGGA	152
			R: AACGAGATGGTATCGGACTTG	
Solute Carrier Family 1 member 4	*SLC1A4* ^a^	NM_001081577.1	F: ATCTTGATAGGCGTGGTTTC	132
			R: GCAACACTGGTTCTCTCTATAA	
Solute Carrier Family 1 member 5	*SLC1A5* ^a^	NM_174601.2	F: TCCAAATCTGCCCAGTCCTCAACT	141
			R: TTCCCATGATTCCCTATGCCCTGA	
Solute Carrier Family 6 member 1	*SLC6A1* ^a^	NM_001077836.1	F: GTGTCTCCATTTCCTGGTTT	150
			R: GCACAGCACTGAAGATGAA	
Solute Carrier Family 6 member 14	*SLC6A14* ^a^	NM_001098461.1	F: GATGCTGCCACACAGATATT	154
			R: TAGCAAATCCAGCGAACAC	
Solute Carrier Family 6 member 6	*SLC6A6* ^a^	NM_174610.2	F: GAAAGCCGTGACGATGAT	158
			R: GGTGAAAGCCCTTCCTTAG	
Solute Carrier Family 7 member 2	*SLC7A2* ^a^	XM_010820288.1	F: GATGCTGGAGGGACTAGAT	146
			R: AGATACCCAGGCACAGAA	
Solute Carrier Family 7 member 4	*SLC7A4* ^a^	NM_001192042.1	F: GACCCACGGACTCTAGTTTA R: GGTTGAGCGATACCTATTGTG	156
Solute Carrier Family 7 member 5	*SLC7A5* ^a^	NM_174613.2	F: TGTGGTCCGATAGGCATAGA	151
			R: ACAACGGTGGATGCTGTT	
Solute Carrier Family 7 member 7	*SLC7A7* ^a^	NM_001075151.1	F: CCTCCAGGTCCTATGTATGT	144
			R: CAGCCAGCAGGAGATAGA	
Solute Carrier Family 7 member 8	*SLC7A8* ^a^	NM_001192889.1	F: GAGATTGGATTGGTCAGTGG	155
			R: GCTCCCACAACTGTGATAAG	
Solute Carrier Family 7 member 11	*SLC7A11* ^a^	XM_010826337.1	F: GTACAGGGATTGGCTTCATC	144
			R: CTGGCACAACTTCCAGTATT	
Solute Carrier Family 17 member 5	*SLC17A5* ^a^	NM_001205974.1	F: CTGCAGTCCCTTATTTAGGC	144
			R: GGAATATCGCAGGTCCAATC	
Solute Carrier Family 17 member 9	*SLC17A9* ^a^	NM_001100378.2	F: GTCTAGACACACCAAGG	141
			R: GGGAAGGTCTCCTTAAA	
Solute Carrier Family 36 member 2	*SLC36A2* ^a^	NM_001206180.1	F: GACGACCAAGGGATAAC	157
			R: AGAGTGGAGGAGATGAA	
Solute Carrier Family 38 member 1	*SLC38A1* ^a^	XM_002687321.3	F: TCACGGTTCGATCTTCTTTATT	131
			R: ATCCTTCATGGAGGGTATGA	
Solute Carrier Family 38 member 4	*SLC38A4* ^a^	NM_001205943.1	F: CTGTGCCCATAGTGCTATTC	139
			R: GGCACAAGGATGACCAAA	
Solute Carrier Family 38 member 6	*SLC38A6* ^a^	XM_010823227.1	F: CACCTCAATACTGCCCATATAC	150
			R: GACGCCACACTGTCATAAA	
Solute Carrier Family 38 member 7	*SLC38A7* ^a^	NM_001100355.1	F: TATAGCTGTGATGGCAAAGG	153
			R: CTCAGGAAGCTGGATATTT	
Solute Carrier Family 43 member 2	*SLC43A2* ^a^	NM_001075546.1	F: AAGGCCCAGGATGAGAT	149
			R: AAGCAGGAGACAGCAAAG	
Cyclophilin	*PPIA* ^b^	NM_178320.2	F: GCCATGGAGCGCTTTGG	65
			R: CCACAGTCAGCAATGGTGATCT	
β-actin	*ACTB* ^#^	NM_173979.3	F: GGATGAGGCTCAGAGCAAGAGA	78
			R: TCGTCCCAGTTGGTGACGAT	
D-aspartate oxidase	*DDO* ^a^	NM_173908.2	F: CTGGCGTATCCAATGTAACC	158
			R: CCTCAAACCCACTTTCTCTC	
Glyceraldehyde 3-phosphate dehydrogenase	*GAPDH* ^b^	NM_001034034.2	F: GCCATCAATGACCCCTTCAT	70
			R: TGCCGTGGGTGGAATCA	
Ribosomal protein S18 Selenocysteine lyase	*RPS18* ^b^ *SCLY* ^a^	NM_001033614.2 NM_001083804.1	F: GCCTGAGAAACGGCTACCAC R: CACCAGACTTGCCCTCCAAT F: AGGAAGGCCAAGGAGATTAT	171 151
			R: CGTGGACTTTGTGGAAGTG	

(F) Primer forward sequence; (R) Primer reverse sequence. ^a^indicates primer sequences obtained by PrimerQuestQM software (IDT technologies).^b^[64]

### Statistical analysis

Cows from each group were ranked according to the plasmatic concentration of P4 at D7, P4 at D7/P4 at D2 ratio, CL size at D7, CL weight, follicle size at D2, D1 and D0 and preovulatory follicle size as previously described [36]. The samples selected for analysis were according to this ranking. Further analyses were conducted in 8 animals per group (D4) and 8 and 9 animals (D7) from the LF-LCL and SF-SCL groups, respectively. The data were tested for normality of residuals using the Shapiro-Wilk test and for homogeneity of variance using the F-max text (SAS; Version 9.2; SAS Institute). The data were analyzed independently for Experiments 1 and 2, as they were not conducted contemporaneously. Discrete dependent variables (diameter of the dominant follicle on D2 and D0, POF diameter, CL volume and weight measured postmortem and plasmatic concentrations of P4 on D4 or D7) were analyzed by one-way ANOVA for the effect of group using the PROC GLM procedure (SAS; Version 9.2; SAS Institute). The amino acid quantification data were tested for the presence of outliers by Dixon’s test, and outliers were removed before the normality of residuals test. Uterine flushing concentrations of amino acid values that did not follow the normality of residuals were transformed for analysis. On D4, uterine flushing concentrations of threonine, alanine, methionine sulfone internal standard, tyrosine, leucine and lysine were transformed to natural log and isoleucine, ornithine and lysine were transformed to rank. On D7, taurine, proline, α-aminobutyric acid, valine, cysteine, isoleucine and lysine were transformed to natural log and β-alanine was transformed to rank. Concentrations of amino acid in uterine flushings and relative gene expression were analyzed by Student’s *t*-test. Means were considered significantly different when they presented a *P* value of 0.05 or less. Means with *P* values between 0.06 and 0.1 were considered as approaching significance.

## Results

### Animal Model

The hormonal strategy that we employed successfully produced groups of cows presenting distinctly different periovulatory ovarian and endocrine characteristics, as expected and described earlier [26, 32, 36–38]. Specifically, for Experiment 1, cows assigned to the LF-LCL group had larger follicle diameters on D2, D1 and D0 compared to animals from the SF-SCL group (*P* < 0.01; Table 2). Pre-ovulatory follicles were also larger for animals from the LF-LCL group (*P* < 0.01; Table 2), but plasmatic concentrations of P4 on D4 were similar for animals from both groups (*P* > 0.05; Table 2). For Experiment 2, cows assigned to the LF-LCL group had larger POF diameters than animals from the SF-SCL group (*P* < 0.01; Table 2). Furthermore, the larger POFs resulted in larger and heavier CLs on D7 (*P* < 0.05; Table 2). Moreover, plasmatic concentrations of P4 on D7 were also greater in cows from the LF-LCL group. More details on ovarian and endocrine responses from the LF-LCL and SF-SCL groups on D4 and D7 were published elsewhere [26, 39].

**Table 2:** Follicle, CL and P4 measurements

End-points	LF-LCL	SF-SCL	*P* <* F*
Experiment 1 (D4) Follicle diameter, mm			
D2	12.66 ± 0.4	8.84 ± 0.6	0.00006
D1	13.93 ± 0.6	9.58 ± 0.3	0.00003
D0	15.64 ± 0.6	11.52 ± 0.4	0.00005
Pre-ovulatory follicle, mm	15.99 ± 0.3	11,32 ± 0.2	0.00000002
CL volume on D4, cm^3^	2.66 ± 1.2	2.3 ± 1	0.03
CL weight on D4, g	1.06 ± 0.06	0.68 ± 0.08	0.002
Plasma P4 concentrations on D4, ng/mL	1.17 ± 0.3	0.8 ± 0.1	0.21
Experiment 2 (D7) Follicle diameter, mm			
D2	10.73 ± 0.7	7.09 ± 0.3	0.0002
D1	11.79 ± 0.6	7.73 ± 0.4	0.000001
D0	13.04 ± 0.5	9.68 ± 0.4	0.00002
Pre-ovulatory follicle, mm	13.63 ± 0.5	10.09 ± 0.3	0.000005
CL volume on D7, cm^3^	2.29 ± 0.2	1.53 ± 0.2	0.03
CL weight on D7, g	2.83 ± 0.2	1.53 ± 0.2	0.0001
Plasma P4 concentrations,ng/mL			
on D7	3.68 ± 0.2	2.1 ± 0.2	0.008

Cows were synchronized to have larger (LF-LCL; Exp 1 *n* = 8; Exp 2 *n* = 8) or smaller (SF-SCL; Exp 1 *n* = 8; Exp 2 *n* = 9) pre-ovulatory follicles and corpora lutea. Mean ± SEM

### Gene Expression

On D4, the abundances of *SLC6A6, SLC7A4, SLC17A5, SLC38A1, SLC38A7* and *SCLY* were on average 64.34, 70.64, 42.36, 56.24, 35,54 and 41.45% greater, respectively, in the endometrium of LF-LCL cows than in the SF-SCL endometrial tissue (*P* ≤ 0.05; Table 3; Fig. 7). These solute carriers are responsible for alanine, serine, proline, taurine, β-alanine, aspartate, glutamate, histidine, ornithine, lysine and glutamine transport. The enzyme SCLY is responsible for alanine synthesis from selenocysteine.

**Table 3:** Relative quantification of transcript abundance by qPCR

Gene	LF-LCL	SF-SCL	*P* < *F*
*SLC1A1*			
D4	0.1215 ± 0.0126	0.1662 ± 0.0339	0.2371
D7	0.0051 ± 0.0004	0.0044 ± 0.0005	0.2830
*SLC1A4*			
D4	0.0107 ± 0.0014	0.0073	0.08
D7	0.0391 ± 0.004	0.0262 ± 0.004	0.05
*SLC1A5*			
D4	1.25 ± 0.08	1.35 ± 0.1	0.45
D7	0.7 ± 0.07	0.9 ± 0.01	0.51
*SLC6A1*			
D4	0.0006 ± 0.0001	0.0005 ± 0.0001	0.5506
D7	0.4561 ± 0.0350	0.2633 ± 0.0282	0.0006
*SLC6A6*			
D4	0.0181 ± 0.0017	0.0110 ± 0.0007	0.0020
D7	0.0682 ± 0.0068	0.0711 ± 0.0090	0.8064
*SLC6A14*			
D4	0.021 ± 0.004	0.025 ± 0.006	0.58
D7	0.0286 ± 0.0028	0.0169 ± 0.0011	0.001
*SLC7A2*			
D4	0.0067 ± 0.0014	0.0068 ± 0.0012	0.9437
D7	0.0047 ± 0.0007	0.0045 ± 0.0009	0.8666
*SLC7A4*			
D4	0.0012 ± 0.0002	0.0007 ± 0.0002	0.052
D7	0.0077 ± 0.0011	0.0034 ± 0.0006	0.0036
*SLC7A5*			
D4	0.0522 ± 0.008	0.0549 ± 0.005	0.78
D7	0.0598 ± 0.01	0.0599 ± 0.007	0.99
*SLC7A7*			
D4	0.0167 ± 0.0012	0.0159 ± 0.0015	0.6886
D7	0.0579 ± 0.0031	0.0434 ± 0.0025	0.0021
*SLC7A8*			
D4	0.0153 ± 0.0021	0.0180 0.0051	0.6238
D7	0.1068 ± 0.0157	0.0373 ± 0.0071	0.0012
*SLC7A11*			
D4	0.0004 ± 0.0001	0.0004 ± 0.0001	0.7969
D7	0.0004 ± 0.0001	0.0005 ± 0.0001	0.8120
*SLC17A5*			
D4	0.0193 ± 0.0012	0.0135 ± 0.0015	0.0089
D7	0.0629 ± 0.0074	0.0362 ± 0.0024	0.0039
*SLC17A9*			
D4	0.0033 ± 0.0005	0.0028 ± 0.0007	0.6243
D7	0.0054 ± 0.0008	0.0048 ± 0.0009	0.6354
*SLC36A2*			
D4	0.0005 ± 0.0001	0.0006 ± 0.0002	0.55
D7	0.0035 ± 0.0013	0.0025 ± 0.001	0.56
*SLC38A1*			
D4	0.066 ± 0.008	0.042 ± 0.004	0.019
D7	0.63 ± 0.08	0.38 ± 0.04	0.01
*SLC38A4*			
D4	0.0072 ± 0.0009	0.0086 ± 0.004	0.19
D7	0.0158 ± 0.009	0.01978 ± 0.002	0.12
*SLC38A6*			
D4	0.0044 ± 0.0001	0.0047 ± 0.0003	0.3745
D7	0.0077 ± 0.0005	0.0066 ± 0.0004	0.1304
*SLC38A7*			
D4	0.0404 ± 0.004	0.0302 ± 0.0014	0.02
D7	0.9601 ± 0.009	0.7069 ± 0.008	0.05
*SLC43A2*			
D4	0.6539 ± 0.0217	0.6357 ± 0.1383	0.8983
D7	0.0065 ± 0.0006	0.0044 ± 0.0006	0.0326
*DDO*			
D4	0.0113 ± 0.0021	0.0119 ± 0.0023	0.8501
D7	0.0208 ± 0.0028	0.0123 ± 0.0011	0.0123
*SCLY*			
D4	0.0090 ± 0.0006	0.0064 ± 0.0003	0.0009
D7	0.0122 ± 0.0011	0.0110 ± 0.0005	0.3279

Mean ± standard error of the mean of the relative abundance of target genes and endogenous controls for large follicle-large CL group (LF-LCL; Exp 1 *n* = 8; Exp 2 *n* = 8) and small follicle-small CL group (SF-SCL; Exp 1 *n* = 8; Exp 2 *n* = 9)

On D7, the transcript abundances of *SLC1A4, SLC6A1, SLC6A14, SLC7A4, SLC7A7, SLC7A8, SLC17A5, SLC38A1, SLC38A7, SLC43A2* and *DDO* were 49.62, 73.26, 68.98, 126.14, 33.32, 186.4, 73.88, 67.93, 35.91, 46.28, and 69.64% greater in the endometrium of the LF-LCL group (*P* ≤ 0.05; Table 3; Fig. 7).

### Amino Acid Quantification in the Uterine Washings

Glutamate, aminoadipic acid, asparagine, serine, glutamine, glycine, histidine, β-alanine, taurine, β-aminoisobutyric acid, threonine, alanine, proline, α-aminobutyric acid, tyrosine, valine, methionine, cystathionine, cysteine, isoleucine, leucine, phenylalanine, tryptophan, ornithine and lysine were the AAs detected in uterine flushings on D4 and D7 (Table 4).

**Table 4:** Concentrations of amino acids in uterine washings

Amino acid	LF-LCL, μmol/g	SF-SCL, μmol/g	*P* < *F*
*GLU*			
D4	115.30 ± 9.23	157.40 ± 23.49	0.066
D7	127.93 ± 13.97	153.13 ± 21.92	0.38
*AAD*			
D4	91.52 ± 0.60	91.63 ± 1.19	0.93
D7	91.54 ± 0.82	92.71 ± 0.93	0.22
*ASN*			
D4	115.63 ± 2.80	126.66 ± 7.56	0.12
D7	139.83 ± 6.55	141.68 ± 6.43	0.84
*SER*			
D4	6.76 ± 1.03	11.04 ± 2.80	0.19
D7	17.34 ± 1.96	15.15 ± 3.12	0.63
*GLN*			
D4	95.74 ± 2.51	108.58 ± 7.08	0.06
D7	120.05 ± 6.16	122.52 ± 7.91	0.81
*GLY*			
D4	119.36 ± 12.39	164.37 ± 31.04	0.13
D7	339.70 ± 41.51	352.53 ± 43.62	0.84
*HYS*			
D4	11.21 ± 0.51	12.81 ± 0.61	0.08
D7	12.42 ± 0.74	12.75 ± 0.82	0.80
*B-ALA*			
D4	70.44 ± 0.60	71.18 ± 0.82	0.58
D7	80.95 ± 3.88	73.78 ± 1.06	0.08
*TAU*			
D4	117.20 ± 9.57	197.90 ± 32.56	0.01
D7	460.47 ± 106.32	334.93 ± 41.22	0.69
*THR*			
D4	6.88 ± 2.00	8.77 ± 2.79	0.66
D7	10.10 ± 1.65	9.83 ± 1.73	0.92
*ALA*			
D4	55.59 ± 5.42	95.70 ± 21.07	0.04
D7	76.94 ± 8.97	91.15 ± 11.40	0.36
*PRO*			
D4	6.27 ± 0.98	9.72 ± 2.21	0.13
D7	29.23 ± 7.78	23.63 ± 3.93	0.81
*AAAB*			
D4	19.82 ± 1.38	28.71 ± 2.85	0.03
D7	32.48 ± 7.75	23.29 ± 2.38	0.82
*TYR*			
D4	6.22 ± 0.99	8.37 ± 1.99	0.16
D7	6.20 ± 0.74	6.77 ± 0.78	0.62
*VAL*			
D4	19.11 ± 1.91	23.90 ± 3.56	0.23
D7	33.11 ± 7.81	16.73 ± 2.27	0.03
*MET*			
D4	22.16 ± 0.26	22.36 ± 0.29	0.64
D7	22.73 ± 0.56	22.70 ± 0.26	0.96
*CYS*			
D4	127.83 ± 6.44	142.07 ± 10.47	0.27
D7	309.37 ± 49.54	177.92 ± 17.55	0.02
*ILE*			
D4	18.09 ± 2.25	21.58 ± 3.25	0.21
D7	15.40 ± 0.95	15.80 ± 1.13	0.93
*PHE*			
D4	120.92 ± 16.94	120.33 ± 25.84	0.98
D7	117.49 ± 23.60	142.63 ± 16.80	0.39
*TRP*			
D4	388.73 ± 35.18	433.83 ± 76.04	0.54
D7	403.82 ± 32.67	398.52 ± 33.38	0.91
*ORN*			
D4	98.07 ± 1.86	96.62 ± 2.29	0.51
D7	97.36 ± 2.33	96.71 ± 1.83	0.82
*LYS*			
D4	4.61 ± 1.04	4.90 ± 1.58	0.65
D7	13.44 ± 3.69	6.73 ± 0.42	0.70

Mean ± standard error of the mean of the concentration of amino acid in μmol/g of uterine washings for large follicle-large CL group (LF-LCL; Exp 1 *n* = 12; Exp 2 *n* = 9) and small follicle-small CL group (SF-SCL; Exp 1 *n* = 7; Exp 2 *n* = 10)

**Fig. 2:**
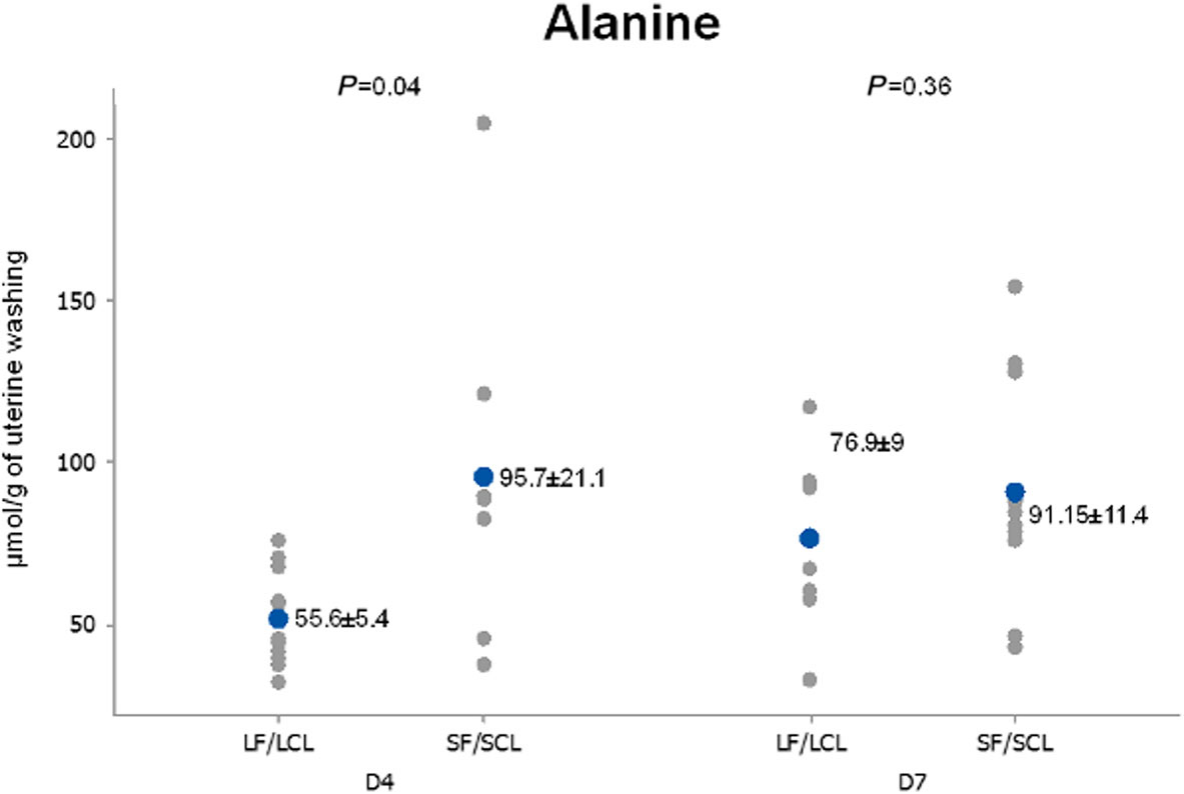
Individual and mean concentrations of alanine in uterine washings from D4 and D7 of diestrus. Each *gray dot* indicates an individual animal. *Blue dots* indicate mean ± sem. LF-LCL indicates Large Follicle-Large CL group and SF-SCL indicates Small Follicle-Small CL group

**Fig. 3:**
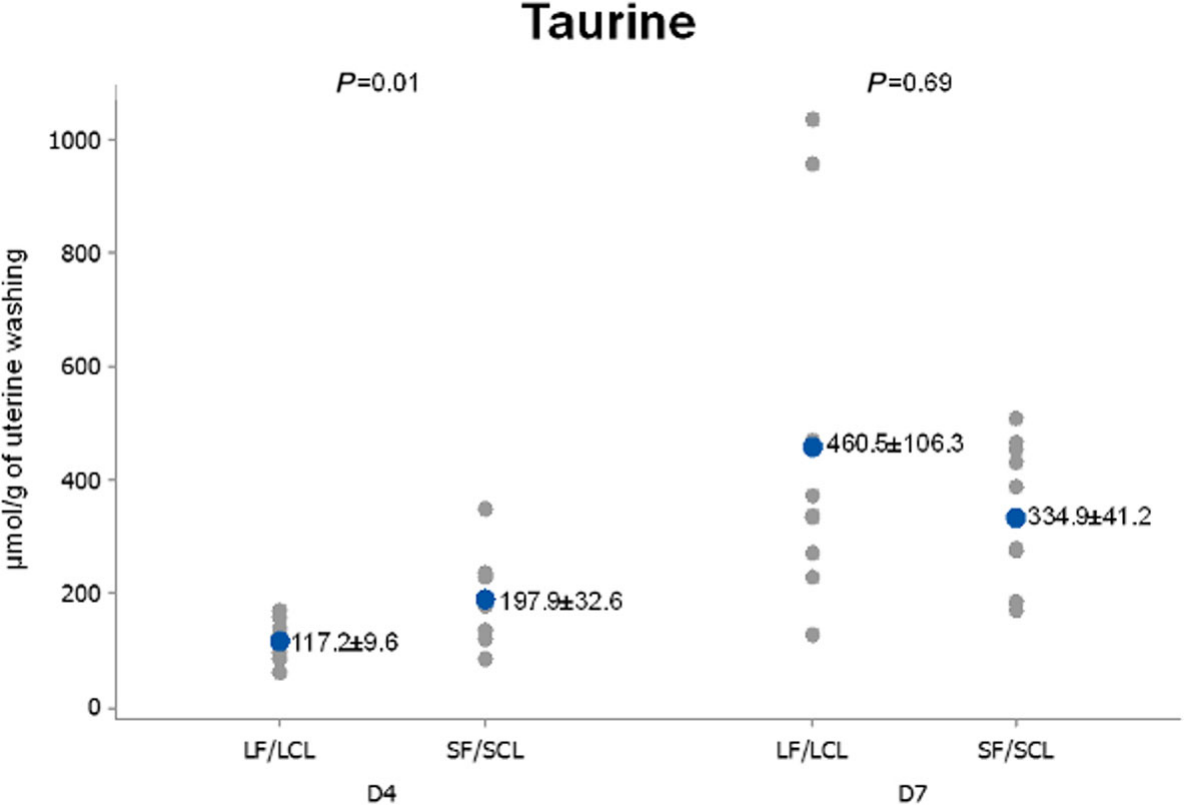
Individual and mean concentrations of taurine in uterine washings from D4 and D7 of diestrus. Each *gray dot* indicates an individual animal. *Blue dots* indicate mean ± sem. LF-LCL indicates Large Follicle-Large CL group and SF-SCL indicates Small Follicle-Small CL group

**Fig. 4:**
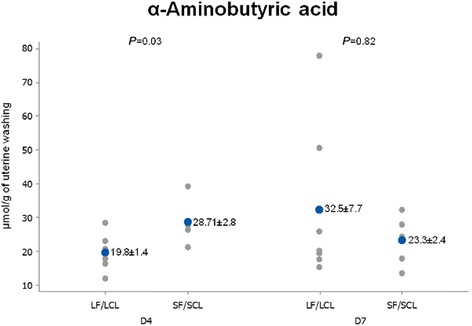
Individual and mean concentrations of α-aminobutyric acid in uterine washings from D4 and D7 of diestrus. Each *gray dot* indicates an individual animal. *Blue dots* indicate mean ± sem. LF-LCL indicates Large Follicle-Large CL group and SF-SCL indicates Small Follicle-Small CL group

When the levels in the uterine washings were compared between groups, D4 data showed that concentrations of taurine, alanine and α-aminobutyric acid in uterine flushings were higher (68.71, 70 and 44.81%, respectively) in SF-SCL washings compared to their LF-LCL counterparts (*P* ≤ 0.05; Table 4; Figs. 2, 3, 4 and 7).

**Fig. 5:**
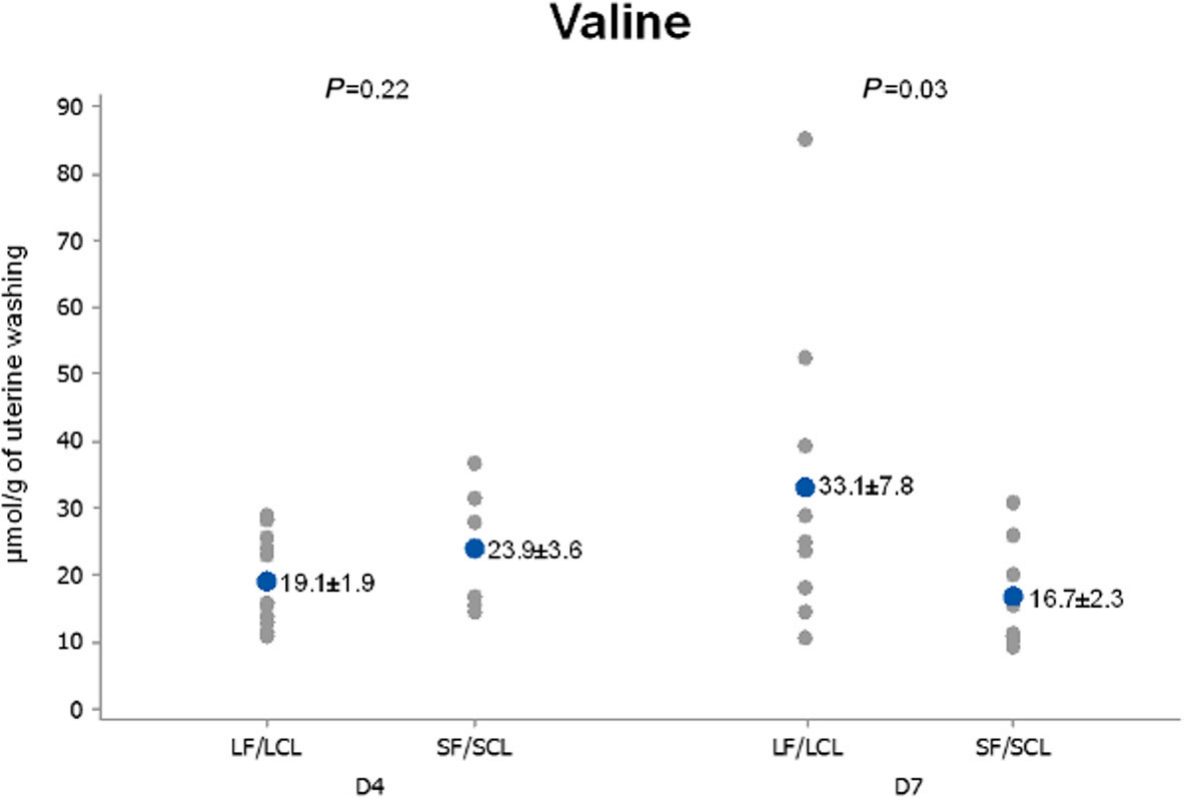
Individual and mean concentrations of valine in uterine washings from D4 and D7 of diestrus. Each *gray dot* indicates an individual animal. *Blue dots* indicate mean ± sem. LF-LCL indicates Large Follicle-Large CL group and SF-SCL indicates Small Follicle-Small CL group

**Fig. 6:**
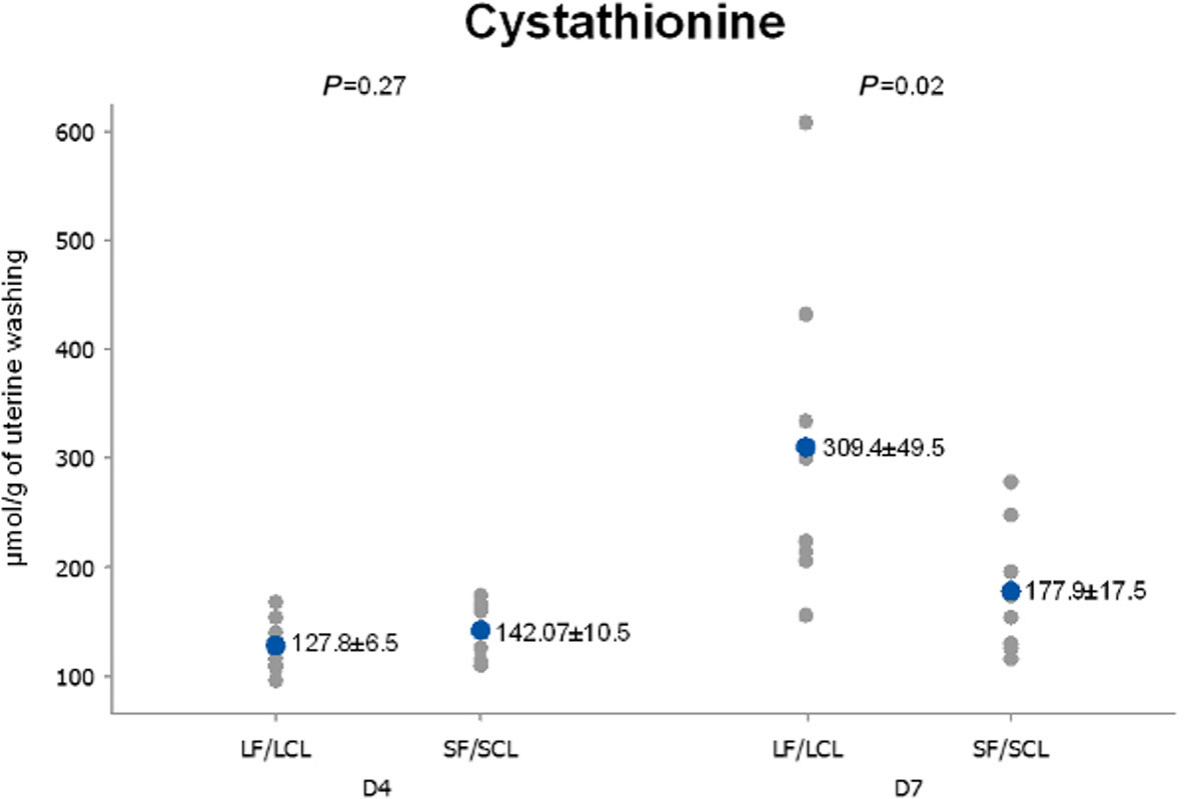
Individual and mean concentrations of cystathionine in uterine washings from D4 and D7 of diestrus. Each *gray dot* indicates an individual animal. *Blue dots* indicate mean ± sem. LF-LCL indicates Large Follicle-Large CL group and SF-SCL indicates Small Follicle-Small CL group

**Fig. 7:**
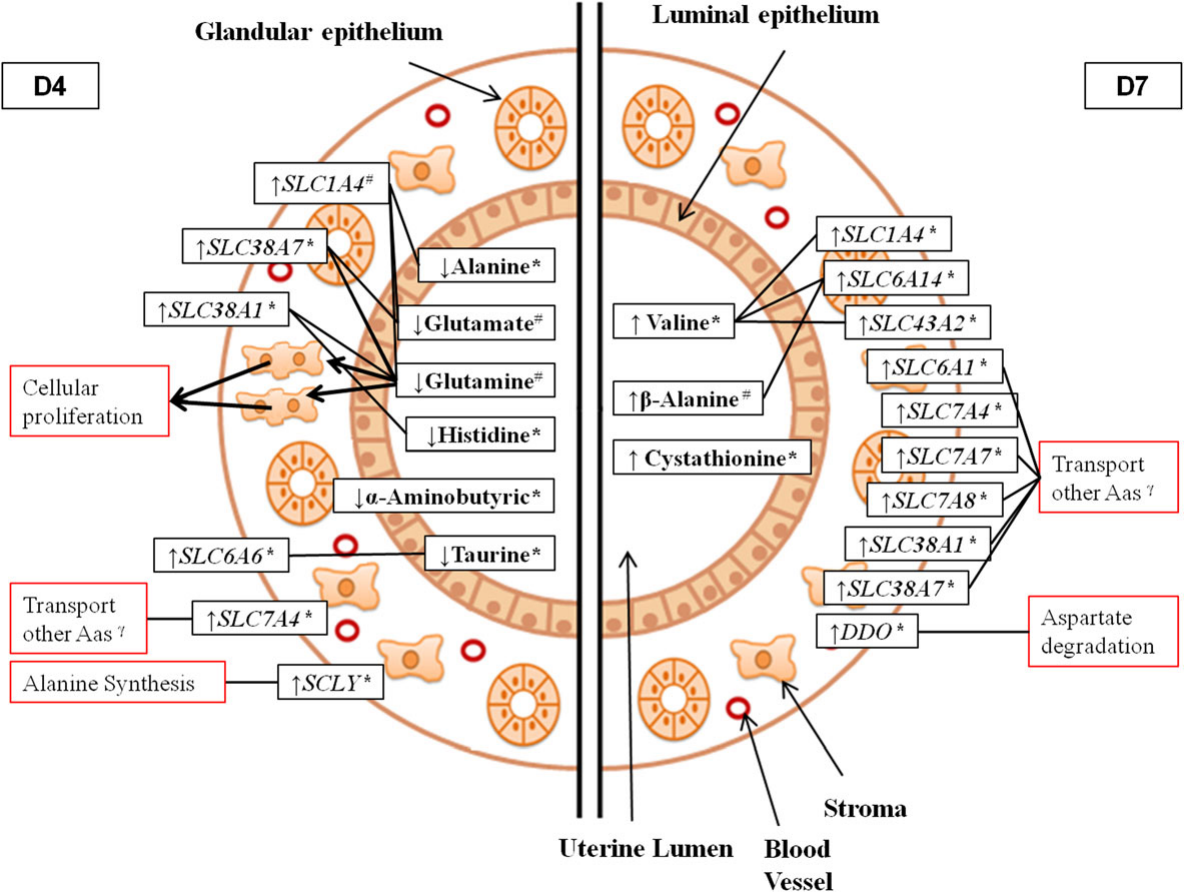
Amino acid transport in the uterus of cows on D4 and D7 of diestrus. This figure shows the comparative abundance of transcripts related to amino acid (AA) transport and metabolism and the luminal concentration of AA between more receptive endometrium (Large Follicle-Large Corpus Luteum group) and less receptive endometrium (Small Follicle-Small Corpus Luteum group) on D4 and D7 after estrus. On D4, the transport of AA seems to occur preferentially from the uterine lumen towards endometrial cells, because despite elevated expression of genes related to AA transporters in endometrium there is lower availability of AA in uterine washings. Such direction of transport benefit events such as cell proliferation, which requires AA. On D7, AA availability in uterine lumen and abundance of genes related to AA transport are both stimulated in the more receptive endometrium. This phenotype is consistent with a greater provision of substrates to support embryonic needs for growth. ↑, up-regulated in LF-LCL group in comparison to SF-SCL group; ↓ down-regulated in LF-LCL group in comparison to SF-SCL group; γ, carriers related to transport of AAs similarly abundant in the lumen of both groups. solid lines connect a transporter with its cognate substrate(s); *, *P* ≤ 0.05; #, *P* < 0.1; *SLC*, Solute carrier protein; *SCLY,* Selenocysteine Lyase; *DDO*, D-aspartate Oxidase

Conversely, on D7, the concentrations of valine and cystathionine in uterine flushings were greater (95 and 74.18%, respectively) in the LF-LCL uterine washings (Table 4; Figs. 5, 6 and 7).

## Discussion

Disappointing fertility success in the beef cow industry is mainly caused by excessive rates of embryonic mortality during early pregnancy, which has been linked to inadequate endometrial receptivity [40, 41]. During early pregnancy, the mother needs to provide the optimal uterine microenvironment for the embryo in order to facilitate initial embryonic-maternal interactions leading to subsequent implantation [42]. However, the exact definition of a microenvironment optimal for the proper development of embryos during early pregnancy remains to be elucidated. As previous studies pointed towards the importance of greater concentrations of AA in the uterus to increase fertility outcome in dairy cows [9], this study attempted to investigate the link between AA metabolic pathways and uterine function to support embryo growth during early diestrus in beef cows. Therefore, we characterized AA transport and metabolic pathways in the endometrium and AA levels in the uterine lumen on D4 and D7 after induction of ovulation in response to different endocrine profiles. Furthermore, the study was conducted in cyclic, non-inseminated cows based on the previous report by Forde et al. [43]. In that report, the authors indicated that from the first week of diestrus until after D13, the pregnant cow endometrium undergoes molecular changes similar to those of the cyclic cow endometrium. Thus, because sample collection was conducted on D4 and D7, it is reasonable to assume that the treatment effects observed in the present report would be similar were the animals pregnant.

In this context, a previously described in vivo receptivity model was used here [26, 32, 36, 38], aiming to define the AA signatures of the receptive endometrial tissues and histotroph. Using the same model, we were able to manipulate two fundamental aspects of receptivity: compared to the LF-LCL group, fertility was lower in the SF-SCL group (i.e., the low receptivity group) [27], and the concentration of PGR in the endometrium was greater in the same group on D7 of the estrous cycle [37]. More specifically, data on the bovine endometrial tissue transcriptome associated with AA transport have been integrated with information on uterine flushing AA profiles from highly receptive (LF-LCL) versus low-receptive (SF-SCL) groups of cows. In beef cattle, altered endocrine patterns during follicle growth are known to influence the follicular size and steroidogenic capacity before ovulation [44]. Thereby, corpora lutea originating from larger follicles are characterized by larger sizes, resulting in greater circulating concentrations of P4 compared to those originating from smaller follicles [45, 46]. In our study, POF influenced CL size and plasmatic levels of P4 on D7 but not on D4. It is important to mention that CL is a transient endocrine organ that starts to develop after ovulation, when theca and granulosa cells differentiate into luteal cells [47]. The D4 and D7 CL are considered to be in different stages of development [48, 49]. Specifically, at D4, the CL is under early development and did not achieve a large enough area to show differences in P4 production as it did on D7. Interestingly, gene expression related to P4 production indicated a greater capacity for P4 production at D7 than at D4 [49]. In cattle, POF size is directly related to E2 production [50, 51], suggesting that POF size and probably circulating concentrations of E2 influence the uterine environment and endometrial gene expression on D4 and D7.

The uterine flushing AA signatures show that on D4, concentrations of taurine, alanine and α-aminobutyric acid in uterine luminal flushings were greater in the SF-SCL group, which in this animal model represents the low-receptive uterus. The mRNA of *SCLY*, the enzyme related to alanine production, was up-regulated in the LF-LCL group. Moreover, *SLC6A6* mRNA, which transports β-alanine and mainly taurine, was more abundant in the endometrium of cows from the LF-LCL group. A collective interpretation of these data suggests that (1) alanine biosynthesis was stimulated in the LF-LCL group and that (2) alanine transport was stimulated in the LF-LCL group, mainly in a lumen-to-endometrium direction. Stimulating accumulation of alanine in the endometrium may be important for endometrial cellular functions, such as proliferation, to guarantee endometrial receptivity. Alternatively, in accordance with data on cardiomyocytes, excess supplementation of AA may downregulate their transporters [52]. This relationship could explain the findings in the SF-SCL group.

Although early bovine embryos are purported to use all available AAs, alanine is unique in that it is not used and is even secreted by the embryo [53]. This suggests that regulation of this AA, which is available in greater concentrations in low-receptive than in high-receptive uteri, plays no major role in early embryonic development. Taurine, which was more abundant in the SF-SCL group, seems to be beneficial for embryonic development [54]; however, the addition of an elevated dose of cysteamine, a taurine precursor, to embryo culture media was toxic to the bovine embryo [55]. Supplementation of α-aminobutyric acid in the embryo culture medium did not affect porcine embryo cell viability [56].

Similar to the alanine transporter, *SLC38A1* is a transporter with high affinity for glutamine and is more abundant in high-receptive endometrial tissue than in low-receptive tissue, whereas glutamine tended to be more abundant in uterine washings of the low-receptive group. These data can be associated with the major proliferative activity found in the LF-LCL endometrium on D4, which was related to adequate uterus conditioning for receiving the embryo [39]. This proliferative activity may also be responsible for the observation of an increase in transporter abundance without an associated increase in the concentration of the target substrate in the uterine lumen. Indeed, glutamine is essential for cell proliferation in other tissues [57].

On D7, regulation of AA transporter transcript abundance and cognate substrates is complex, and several scenarios were observed. For example, *SLC1A4* was more abundant in the endometrium of LF-LCL cows, although its direct targets, serine, tyrosine, threonine, were present in similar concentrations in the uterine lumen of cows of both groups. Likewise, *SLC38A1* and *SLC38A7* transcripts were up-regulated in the endometrium of the LF-LCL group on D7. These transporters have high affinity for glutamine transport, but this AA was present in similar concentrations in the uterine lumen of both groups. In contrast, regardless of a similar abundance of the transporter *SLC7A11* between groups on D4 and D7, cystathionine, a product of serine and homocysteine condensation by cystathionine β-synthase [58], and valine were present in greater concentrations in the uterine washings of cows from the LF-LCL group compared to cows from the SF-SLC group. In a third scenario, SLC6A14 and one of its transporting targets, valine, were stimulated in the LF-LCL group. Correspondingly, in the human cervix, an increase in SLC6A14 mRNA was associated with a parallel increase in protein [59]. Interestingly, after the 4-cell stage, valine, along with leucine, isoleucine, and methionine, was favorable for the cleavage rates of the diploids after compaction and increased the total number of cells in the blastocyst and inner cell mass [60]. SLC7A8 transcript was up-regulated in the LF-LCL endometrium on D7, and this carrier transports neutral AAs such as valine, which is also up-regulated in the LF-LCL group. *SLC7A8* is also responsible for alanine transport. Despite the fact that the concentration of alanine in uterine washings is similar for both groups, the transcript abundance of *DDO*, an enzyme related to alanine degradation, is up-regulated in LF-LCL group. Perhaps there is a requirement to strictly control luminal concentrations of alanine on D7.

In this study, AA concentrations were quantified using uterine washings, composed of histotroph diluted in 20 mL of PBS. Such a method of quantification is appropriate for estimating AA availability in the uterus.

Collectively, the present data suggest that in the more receptive uterine phenotype, on D4, AA transport and metabolic pathways are directed to supply endometrial requirements for growth and function. Coincidentally, around D4, embryos have a limited demand for AAs, and they are in transit from the oviduct to the uterus. Thus, the AA composition of the histotroph reflects the endometrial demands for proper function more than a milieu for embryonic development. More interestingly, on D7, when the embryo is expected to be in the uterus, AAs are more abundant in the uterine secretions of the LF-LCL group, probably to serve as a supply to meet embryonic requirements. Correspondingly, transcript abundance data show a greater number of AA transporters that are up-regulated in the more receptive endometrium on D7. This phenotype is probably associated with the greater AA requirement for the subsequent stages of embryonic development (i.e., hatching and elongation). In terms of sex steroid regulation of such processes, the phenotype on D4 reflects classical actions of estradiol that include stimulation of endometrial cell proliferation, while the phenotype on D7 is associated with actions of progesterone, such as glandular secretory activity and histotroph formation to support embryonic growth [4, 39, 61–63]. A summarized view of the AA transport systems in the endometrium in association with receptivity during early diestrus is presented in Fig. 7.

To the best of our knowledge, this is the first investigation that relates early diestrus temporal changes in AA transport and availability in the uterine lumen to different periovulatory endocrine patterns that characterize fertility phenotypes. This study points to an important link between AA metabolic pathways in the endometrium and uterine receptivity. In addition, these data can serve as a basis for novel studies in bovine reproduction biotechnology with the aim of developing new tools to improve beef cattle fertility and profitability.

## Conclusions

This study showed that the transcript abundance of AA transporters in the endometrium is linked with the receptive state of the endometrial tissue at both D4 and D7. Additionally, the AA patterns in uterine flushings differ between contrasting receptivity status of cows, both at D4 and at D7. The latter data indicate that AA metabolism and transport may be a potential key that needs to be regulated in order to fine-tune the maternal receptive state during early diestrus.

## Abbreviations

AA: Amino acid
AAAB: α-amino butyric acid
AAD: α-amino adipic acid
ALA: Alanine
ANOVA: Analysis of variance
ASN: Asparagine
ASNS: Asparagine synthase
B-ALA: β-alanine
cDNA: Complementar deoxyribonucleic acid
CL: Corpus luteum
CYS: Cysteine
DDO: D-aspartate oxidade
E2: Estradiol
GLN: Glutamine
GLU: Glutamate
GLY: Glycine
GnRH: Gonadotrophin releasing hormone
GPT: Glutamic-pyruvate transaminase
HPLC: High capacity liquid chromatography
HYS: Histidine
ILE: Isoleucine
LF-LCL: Large follicle-large CL group
LYS: Lysine
MET: Methionine
ORN: Ornithine
P4: Progesterone
PBS: Phosphate buffer solution
PHE: Phenylalanine
POF: Pre-ovulatory follicle
PRO: Proline
PROC GLM: General linear models procedure
qRT-PCR: Real-time reverse transcriptase-polymerase chain reaction
RNA: Ribonucleic acid
SCLY: Selenocysteine lyase
SER: Serine
SF-SCL: Small follicle-small CL group
SLC: Solute carrier protein
TAU: Taurine
THR: Threonine
TRP: Tryptophan
TYR: Tyrosine
VAL: Valine
